# Activation of MAPK ERK in peripheral nerve after injury

**DOI:** 10.1186/1471-2202-7-45

**Published:** 2006-06-08

**Authors:** S Agthong, A Kaewsema, N Tanomsridejchai, V Chentanez

**Affiliations:** 1 Department of Anatomy, Faculty of Medicine, Chulalongkorn University, Rama IV Road, Pathumwan, Bangkok, 10330, Thailand

## Abstract

**Background:**

Activation of extracellular signal-regulated protein kinase (ERK), a member of mitogen-activated protein kinase (MAPK) family, has been proposed to mediate neurite outgrowth-promoting effects of several neurotrophic factors *in vitro*. However, the precise activity of ERK during axonal regeneration *in vivo *remains unclear. Peripheral axotomy has been shown to activate ERK in the cell bodies of primary afferent neurons and associated satellite cells. Nevertheless, whether ERK is also activated in the axons and surrounded Schwann cells which also play a key role in the regeneration process has not been clarified.

**Results:**

Phosphorylation of ERK in the sciatic nerve in several time-points after crush injury has been examined. Higher phosphorylation of ERK was observed in the proximal and distal nerve stumps compared to the contralateral intact nerve from one day to one month after crush. The activation of ERK was mainly localized in the axons of the proximal segments. In the distal segments, however, active ERK was predominantly found in Schwann cells forming Bungner's bands.

**Conclusion:**

The findings indicate that ERK is activated in both the proximal and distal nerve stumps following nerve injury. The role of activated ERK in Wallerian degeneration and subsequent regeneration *in vivo *remains to be elucidated.

## Background

Peripheral axotomy can activate several signaling pathways in neurons and associated glial cells leading to two opposing consequences: cell death or adaptation to regenerate neurites. The principal signaling pathways that have been demonstrated to be involved in axonal regeneration are PI3K-Akt [1-3] and JAK/STAT [4,5].

Accumulated evidence has shown the participation of mitogen-activated protein kinases (MAPKs) during axonal injuries. MAPKs are a family of serine/threonine specific kinases that transduce extracellular stimuli to altered gene expression and have been shown to play a role in diverse cellular events ranging from proliferation, differentiation to apoptosis. So far, three subfamilies of MAPKs have been identified, namely: extracellular signal-regulated kinase (ERK), c-Jun NH_2_-terminal kinase (JNK) and p38 kinase (p38). JNK along with its main transcription factor, c-Jun, are activated in the dorsal root ganglia (DRG) after sciatic nerve transection [6-8]. Similarly, it has been reported that p38 was activated in axotomized neurons and, in case of spinal root ligation, this activation has been linked to the development of mechanical allodynia [9-11]. Moreover, inhibition of p38 leads to enhanced axonal regeneration, suggesting that p38 is likely involved in this process [12].

As for ERK, its role in neurite extension in response to growth factors is well-recognized. Following axotomy, ERK activation has been observed in transected sciatic nerve and ipsilateral DRG [4,13]. Evidence suggests that this increased activity of ERK is mediated by endogenous neurotrophic factors known to stimulate axonal regeneration. Nerve growth factor (NGF) which is known to be up-regulated shortly after nerve injury, requires ERK to promote neurite outgrowth *in vitro *[14,15]. Moreover, other regeneration-promoting molecules, such as glial-derived neurotrophic factor (GDNF) and FK506 also mediate their effects via the ERK pathway [15,16]. Therefore, it is possible that activation of ERK is essential for axons to regenerate in response either to endogenous growth factors or exogenous molecules. Although the ERK activation has been linked to mechanical allodynia in the models of neuropathic pain [18,25], the precise function of activated ERK after nerve injury *in vivo*, especially in term of axonal regeneration, is still unclear. In fact, spatial and temporal changes of ERK activity in the peripheral nerve in response to injuries have not yet been studied. Hence, the objective of this work was to investigate the time-dependent changes in the activation of ERK in crushed sciatic nerve with correlation to the cell types.

## Results

### Activation of ERK in sciatic nerve after crush

**Figure 1:**
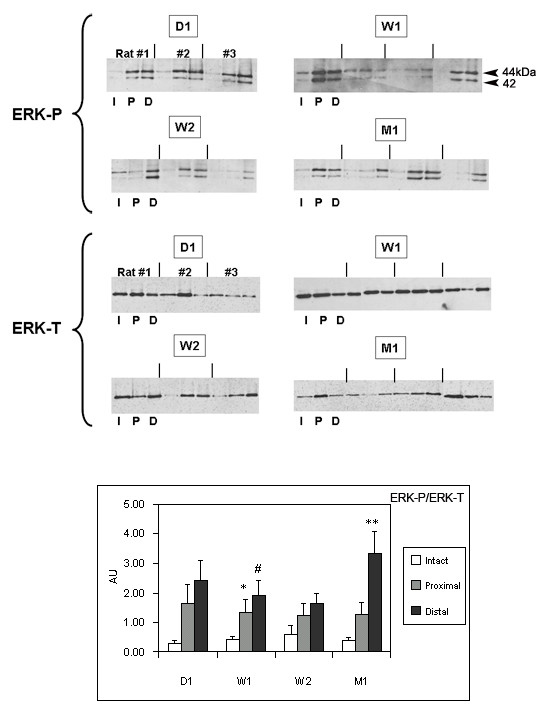
Phosphorylation of ERK in sciatic nerves at various time points after nerve crush. Sample immunoblots probed for ERK-P and ERK-T are shown above. For ERK-P, two isoforms were observed (ERK1 at 44 kDa and ERK2 at 42 kDa), whereas they were not distinguishable in ERK-T. The bar chart below demonstrates the ratio of ERK-P to ERK-T in three nerve segments for each time point. The data are means ± SEM. AU = arbitrary unit, D1 = day 1, W1 = week 1, W2 = week 2, M1 = month 1, I = intact nerve, P = proximal segment to the crush lesion, D = distal segment to the crush lesion. * p < 0.05 vs. W1 intact, # p < 0.01 vs. W1 intact, ** p < 0.05 vs. M1 proximal and p < 0.01 vs. M1 intact, * and # by Kruskal-Wallis test, ** by ANOVA, n = 6–7 for each time-point.

Levels of phosphorylated ERK (ERK-P) relative to those of total ERK (ERK-T) were elevated in the proximal nerve segment compared to the intact nerve in all time-points with statistically significant changes observed at week1 after crush (Figure 1). In the distal stump, ERK phosphorylation was even higher relative to the proximal nerve, especially at post-crush 1 week and 1 month. No differences in the expression of ERK (ERK-T) were found among various nerve segments from any time point (quantitative data not shown).

### ERK activation in axons and Schwann cells

**Figure 2:**
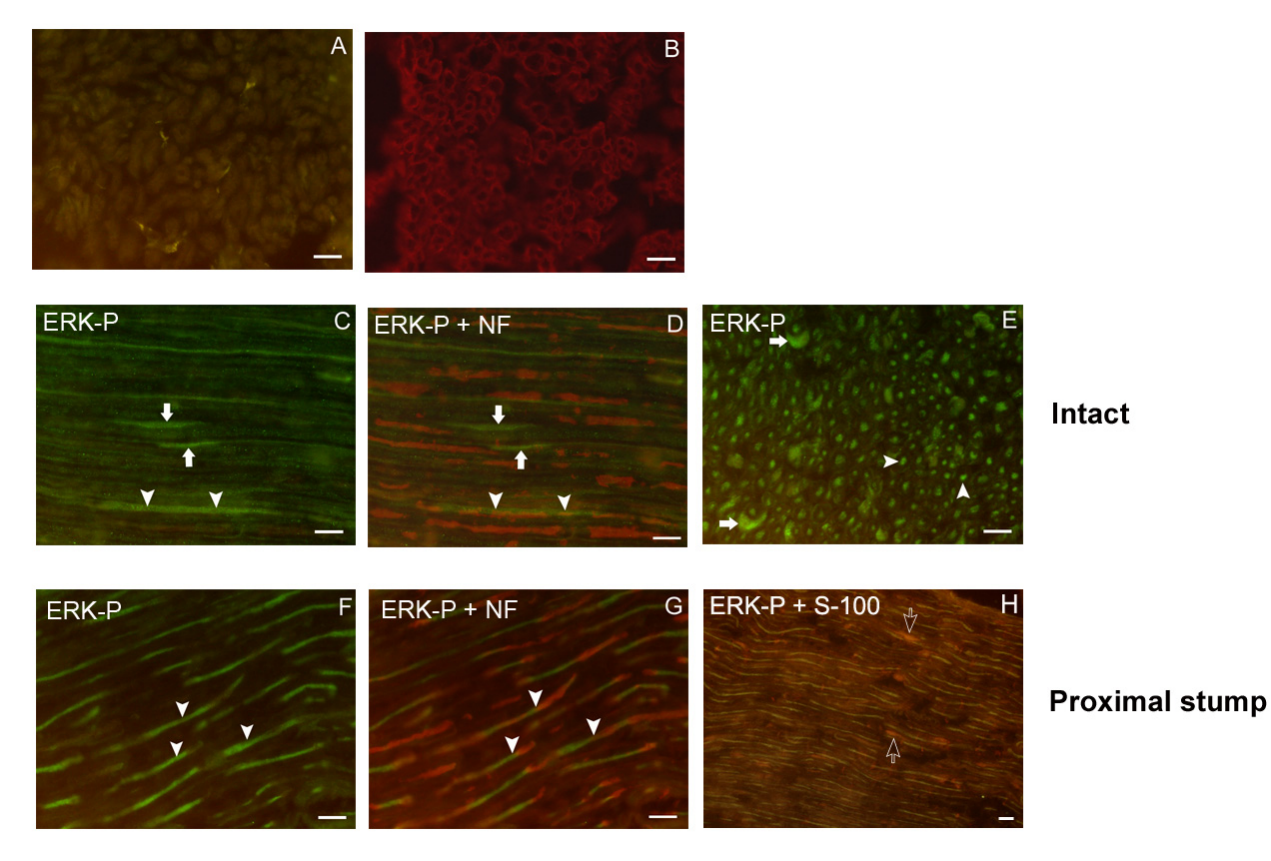
Immunoreactivity of phospho-ERK (ERK-P) in the intact and proximal stumps of crushed sciatic nerves. The nerves were longitudinally sectioned (C, D, F, G and H) or transversely sectioned (A, B and E). The sections exposed to secondary antibodies conjugated with FITC or rhodamine without primary antibodies are shown in A and B, respectively. C-E were from intact nerves. F-H were representatives of the proximal stumps from all time points studied. Single staining for ERK-P only (C, E and F). Double staining for ERK-P (green) and pan-neurofilament (NF, red) (D & G). Double labeling for ERK-P (green) and S-100 (red) (H). Arrowheads indicate the locations of axons; filled arrows indicate the ERK-P immunoreactivity in Schwann cells. In the intact nerve, ERK-P was present in the axons as indicated by co-localization with pan-neurofilament (C & D), and Schwann cells as recognized by the characteristic semilunar shape (E). In the proximal stump, ERK-P was exclusively co-localized with pan-neurofilament in the axons (F & G), but not Schwann cells (open arrows in H). Scale bars represent 40 μm.

**Figure 3:**
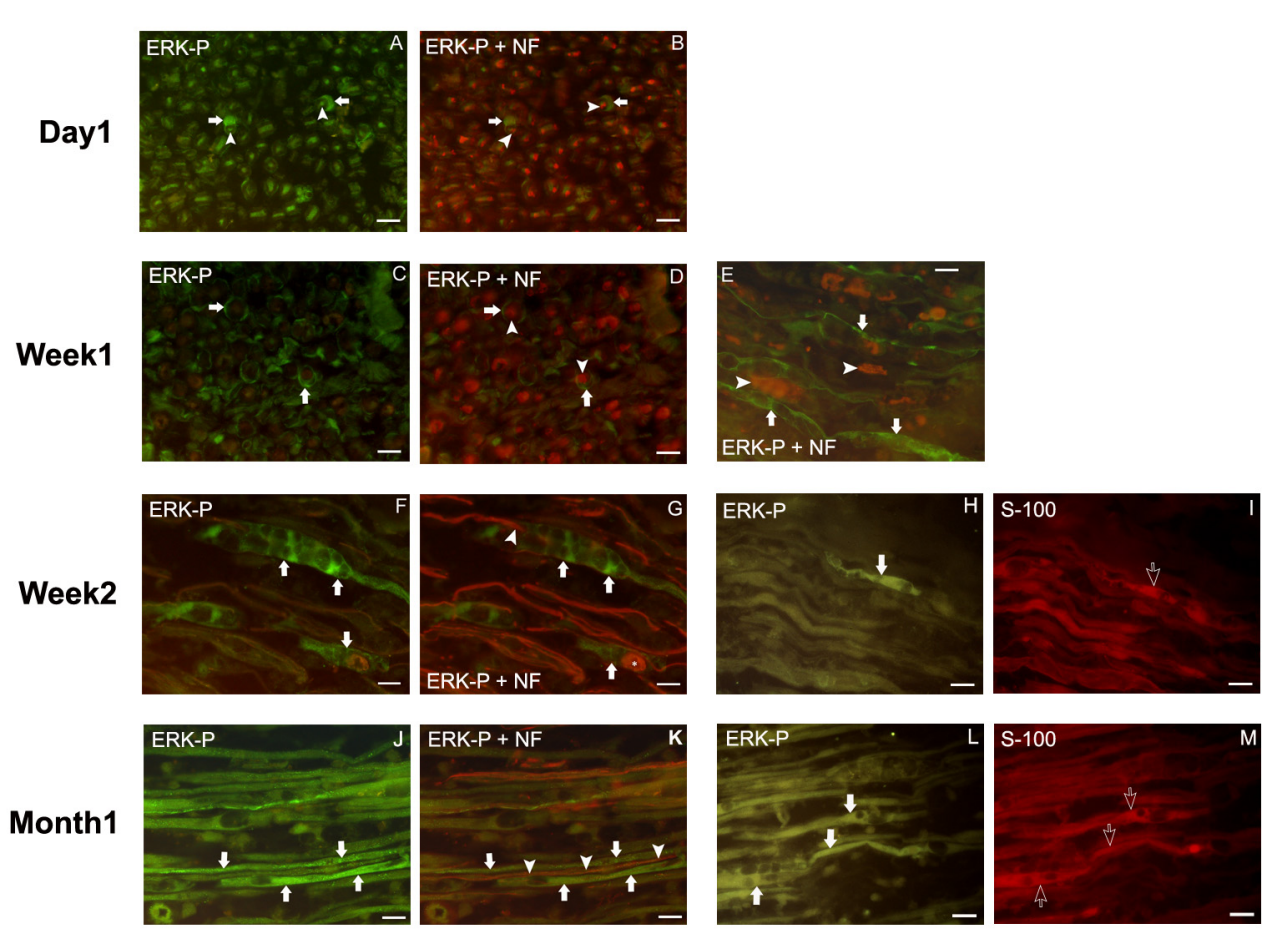
Immunoreactivity of ERK-P in the distal stumps of sciatic nerves in various time points after crush injury. Longitudinal sections are shown in E-M. Transverse sections are shown in A-D. Single staining for ERK-P only (A, C, F, H, J and L). Single staining for S-100 (red) (I and M). Double staining for ERK-P (green) and pan-neurofilament (NF, red) (B, D, E, G and K). Arrowheads indicate the locations of axons; filled and open arrows indicate the ERK and S-100 immunoreactivities in Schwann cells, respectively. At post-crush 1 day (A & B), ERK-P immunoreactivity was observed in the axons and with higher frequency in Schwann cells compared to the intact nerve as indicated by partial co-localization of ERK-P with pan-neurofilament. Afterwards, ERK-P was exclusively localized in Schwann cells forming Bungner's bands since it was co-localized with S-100 (H-I and L-M) but not NF at post-crush 1 week (C-E), 2 weeks (F-G) and 1 month (J-K). Scale bars represent 40 μm.

No positive immunoreactivity was observed when primary antibodies were omitted in the presence of either secondary antibodies conjugated with FITC or rhodamine (Figure 2A and 2B). In the intact nerve, the ERK-P immunoreactivity was found in the axons identified by its co-localization with the pan-neurofilament immunoreactivity (Figure 2C–2E). In addition, ERK-P was also located in Schwann cell cytoplasm which appeared as crescent structures encapsulating the axons (Figure 2E). However, ERK-P was present mainly in the axons in the proximal nerve stump from all time points (Figure 2F–2H). In the distal nerve stump at day 1 after crush, ERK-P immunoreactivity was found in axons and with higher frequency in Schwann cells compared to the intact nerve (Figure 3A and 3B). In contrast, from week 1 until month 1, ERK-P was predominantly located between clumps of degenerated axons whose unremoved neurofilament proteins were stained red at week 1 (Figure 3C–3E) and between the regenerated axons at 2 weeks and 1 month post-crush (Figure 3F–3G and 3J–3K, respectively). This location of ERK-P was likely the Bungner's band, the tube forming by Schwann cells, since the co-locatization of ERK-P and S-100 (a Schwann cell marker) immunoreactivities was demonstrated (Figure 3H–3I and 3L–3M).

## Discussion

This study has demonstrated the activation of ERK in the proximal and distal nerve stumps from one day to one month after crush. Immunohistochemistry has shown that this activation occurred mainly in the axons of the proximal nerve, whereas the activation was more prominent in Schwann cells forming Bungner's bands in the distal nerve. Increased phosphorylation of ERK in the injured sciatic nerve has been previously reported by Sheu and co-workers [4]. They found that ERK-P levels were increased in the proximal nerve segment adjacent to the transection site as well as in the distal segment starting from one day until at least 16 days after operation. However, they have not demonstrated in which cell types this activation occurred. In accordance with that study, we found the prolonged activation of ERK from one day to one month after crush with slightly higher degree in the distal stumps compared to the proximal stumps. The expression of ERK as determined by the levels of ERK-T appears to be unaffected by the injury although the high variations can be observed. These high variations of ERK-T along with those of ERK-P may explain the insignificant increase in the phosphorylation ratio of ERK in the crushed nerve compared to the intact nerve at week 2. In addition, for the first time, this study showed that this ERK activation occurred mainly in the axons of proximal nerve. Early in the distal nerve at post-crush 1 day, the active ERK was observed both in the axons and Schwann cells. Furthermore, from week 2 to month 1, ERK-P was almost exclusively expressed in the Bungner's bands of Schwann cells.

The ERK activation in the axons of the proximal stump might be related to its activation in the neuronal cell bodies. The previous study has shown that ERK was activated in spinal ganglion neurons and satellite cells 7 and 14 days after sciatic nerve injury [13]. Interrupted anterograde axonal transport at the crush site resulting in the accumulation of ERK-P in the proximal nerve segment may account for the earlier increase in the expression of ERK-P in this part at post-crush day 1 than in the DRG (7 days). This possibility is supported by the previous report of ERK axonal transport in DRG neurons [23]. At later time points, 1, 2 and 4 weeks post-lesion, elevated ERK-P in the proximal stump may be due to the upregulated ERK phosphorylation in the cell bodies. Whether anterograde transport of ERK-P is involved in the up-regulation of ERK-P in the proximal stump remains to be clarified. It is also worth noting that the ERK-P immunoreactivity was observed in many Schwann cells in the intact nerve, whereas most positive signals in the proximal stump were localized to the axons. This may suggest the down-regulation of ERK-P in Schwann cells in the proximal segment after injury. Nevertheless, the significance of this change needs to be clarified.

The underlying mechanisms that stimulate the ERK pathway are unknown. It is unlikely that ERK was activated by the upregulated cytokines, such as, interleukin-1 beta (IL-1β), interleukin-6 (IL-6) since the patterns of upregulation of these cytokines were not correlated with that of ERK-P [4]. Some evidence shows that the upregulated growth factors after nerve injury may be responsible for this ERK activation in DRG. Obata and colleagues have administered NGF either intrathecally or intraneurally and found an increase in the ERK phosphorylation in L4/5 DRG [13,18]. Furthermore, GDNF, another growth factor whose expression is also upregulated after nerve injury can activate ERK in cultured DRG neurons [15]. It is also noteworthy that ERK-P was observed in satellite cells around neuronal cell bodies. The importance of this finding is not known but may emphasize the role of glial cells or the glial-neuronal interaction in the DRG after nerve injury.

Although the downstream events in the process of axonal regeneration stimulated by the active ERK remain unknown, the importance of ERK activation in nerve regeneration is increasingly evident. In the neuronal cell bodies, at least one study has demonstrated that ERK was required for an upregulated expression of brain-derived neurotrophic factor (BDNF) following axotomy [13] and BDNF can accelerate axonal regeneration [21,22]. Moreover, ERK is likely to mediate the regeneration-promoting effects of NGF *in vitro *[14,15] and *in vivo *[19,20]. Similarly, ERK was required for the neurite outgrowth stimulated by GDNF [15]. Therefore, it appears that the ERK pathway is essential for nerve regeneration.

In the distal nerve stump, Schwann cells proliferate to form Bungner's bands following Wallerian degeneration. This activity may need the ERK activation as one study has found that ERK was required for Schwann cell proliferation induced by ascorbate in the co-culture of DRG neurons and Schwann cells [24]. ERK has also been shown to be involved in Schwann cell proliferation triggered by leprosy bacilli [26]. Furthermore, ERK has been shown to play a role in maintaining Schwann cells in an immature state which can still proliferate by counteracting the effect of PI3K [3]. The main localization of ERK-P in Bungner's bands observed in this study is in accordance with these findings. The responsible mechanisms that stimulate the ERK pathway in these proliferating Schwann cells are not clarified. Nevertheless, Sheu and colleagues have reported that the sustained pattern of ERK-P up-regulation in the distal stump was correlated with three sequential peaks of increased expression of growth factors: NGF, GDNF and BDNF, in chronological order [4]. Whether there is a relationship between these growth factors and ERK phosphorylation during Schwann cell proliferation remains to be studied. Besides the possible role of ERK in Schwann cell proliferation, it has been proposed that ERK may be also involved in the elimination of supernumerary Schwann cells through up-regulated p75^NTR ^in the advanced stage of regeneration [27,28]. Taken together, the above evidence may explain the sustained and more pronounced activation of ERK in the distal nerve.

## Conclusion

The higher phosphorylation of ERK was found in the proximal and distal stumps of sciatic nerve from one day until one month after crush. In the proximal segments, active ERK was mainly localized to the axons, whereas it was exclusively expressed in Schwann cells forming the Bungner's bands in the distal segment. These findings indicate that ERK is also activated in the injured peripheral nerve in addition to in the DRG. To elucidate the precise role of ERK in peripheral nerve regeneration, strategies to inhibit the ERK pathway must be employed and various components participating in the regeneration should be carefully examined.

## Methods

### Animal surgery and sacrifice

Forty male Wistar rats weighing 200–250 g were anesthetized using halothane and underwent unilateral sciatic nerve crush. The left sciatic nerve of each animal was exposed at the mid-thigh level and crush was induced by the use of a fine arterial clamp with firm pressure against the nerve for 30 s. Epineurial suture with Ethilon^® ^6/0 was done to mark the crush site. Following the surgery, the wound was closed and sutured with Ethilon^® ^4/0. The animals were allowed to recover and housed in an animal care unit until sacrifice. All experimental procedures were approved by the institutional ethics committee and were performed according to the guidelines of the National Research Council of Thailand.

Eight rats per each time-point were sacrificed 1 day, 1 week, 2 weeks and 1 month after nerve crush. The sciatic nerves were removed bilaterally and snap-frozen on dry ice. These tissues were later transferred to -70°C and kept there until use for Western blot analysis. Additional 2 rats underwent transcardial perfusion at each time-point with 200 ml of 0.9% NaCl followed by 500 ml of 4% paraformaldehyde (PFA). The sciatic nerves were removed and post-fixed in 4% PFA for 6 hours at 4°C. After fixation, the tissues were washed several times and kept in 20% sucrose in 0.1 M phosphate buffer pH 7.4 until the next process of immunohistochemistry.

### Western blot analysis

The sciatic nerves on the crush side were divided at the crush location marked by the suture into the proximal and distal stumps, whereas the whole contralateral (intact) nerves were used. All nerve segments were homogenized in homogenization buffer [0.1 mmol/l PIPES pH 6.9, 5 mmol/l magnesium chloride, 5 mmol/l EGTA, 0.5% Triton X-100, 20% glycerol, 10 mmol/l sodium fluoride plus 1 mmol/l PMSF, 2 mmol/l sodium orthovanadate and protease inhibitor cocktail (1 μg/ml pepstatin A, 1 μg/ml leupeptin, 10 μg/ml benzoyl-L-arginine methyl ester, 10 μg/ml p-tosyl-L-arginine methyl ester, 10 μg/ml L-1-tosylamide-2-phenylethylchloromethyl ketone, 10 μg/ml trypsin inhibitor and 10 μg/ml aprotinin); all from Sigma]. Sample buffer (0.25 M Tris pH 6.8, 10% glycerol, 0.01% bromophenol blue, 10 mM dithiothreitol, 2% SDS and 2% β-mercaptoethanol; all from Sigma) was added to the samples before boiling for 5 min. These homogenized samples were stored at -20°C until use.

Concentration of protein in each sample was determined using Bramhall protein assay [17]. SDS-PAGE was performed on 10 μg protein in 10% acrylamide and proteins were transferred to nitrocellulose membrane (Hybond ECL, Amersham Biosciences) using a semi-dry electroblotter (Trans-Blot SD semi-dry transfer cell, Bio-Rad). Prevention of non-specific binding on the membrane was achieved by incubating with 10% dried skim milk in Tween buffer (0.05% Tween20, Sigma). The membranes were then incubated overnight at 4°C in primary antibodies [rabbit antibodies to total and phosphospecific ERK1 and 2 (1:500 and 1:5000, respectively, Santa Cruz Biotechnology)]. Different membranes were probed for total and phosphospecific ERK. In the following day, the membranes were washed and incubated in the secondary antibody conjugated with horse-radish peroxidase (HRP) (anti-rabbit-HRP 1:5000, Cell Signaling Technology) for 2 hours at room temperature. The membranes were washed and the immune complex was detected by enhanced chemiluminescence (LumiGLO, Cell Signaling Technology). Hyperfilms (Hyperfilm ECL, Amersham Biosciences) were exposed to the membranes and scanned with a flat-bed scanner. All scanned digital images were imported to microcomputer in tiff format and the densities of specific bands were analyzed with image analysis program (Image ProPlus 4.5). Results from different blots were combined using standards present in every blot in triplicate and are expressed as a ratio of phosphorylated to total protein. The data of 2 isoforms of ERK (ERK1 and ERK2 at 44 and 42 kDa, respectively) were combined and shown in a bar chart.

### Statistical analysis

The data were imported to SPSS for Windows version 10 and checked for normal distribution and homogeneity of variances. If these assumptions were met, one-way analysis of variance (ANOVA) was employed to compare means from different time-points. However, if the measurements were extremely skewed from normal distribution and/or had a markedly significant difference in variances, a non-parametric test (Kruskal-Wallis test) was used instead. Where statistically significant differences were observed in ANOVA or Kruskal-Wallis test, pair-wise or post-hoc comparisons were achieved by using Tukey's HSD or Mann-Whitney U test, respectively. Statistically significant differences were considered when p < 0.05 unless otherwise stated.

### Immunohistochemistry

Sciatic nerves kept in 20% sucrose in PBS were embedded in OCT medium and 9 μm-thick slices were cut by cryostat section. Proximal and distal segments of sciatic nerves were sectioned either transversely or longitudinally. The slides with sections were blocked in 10% normal serum (Sigma) and incubated in the primary antibody to phosphorylated ERK (1:200, Cell Signaling Technology) for 48 hours at 4°C. After washing, the slides were incubated in the secondary antibody conjugated with fluorescein isothiocyanate (FITC) (anti-rabbit-FITC 1:200, Santa Cruz Biotechnology) for 2 hours at room temperature. The slides were then mounted with anti-fading mounting medium (Vectashield, Vector Laboratories), cover-slipped and examined under fluorescence microscope. In some sections, double-staining with either anti-pan-neurofilament (1:50, Zymed) or anti-S-100 (1:100, Chemicon) antibodies overnight at 4°C followed by secondary antibody conjugated with rhodamine (1:200, Santa Cruz Biotechnology) was done to locate the axons or Schwann cells, respectively.

## Authors' contributions

SA carried out the Western blot and immunohistochemical experiments, analysis of the data and drafting the manuscript.

AK and NT carried out the animal experiment and collection of tissues.

VC participated in the design of the study, gave advise during the experiments and correcting the manuscript.
